# ASXL1 as a critical regulator of epigenetic marks and therapeutic potential of mutated cells

**DOI:** 10.18632/oncotarget.26230

**Published:** 2018-10-16

**Authors:** Daichi Inoue, Takeshi Fujino, Toshio Kitamura

**Affiliations:** Daichi Inoue: Human Oncology and Pathogenesis Program, Memorial Sloan Kettering Cancer Center, Zuckerman, New York, NY, USA

**Keywords:** MDS, ASXL1, H3K4me3, H2AK119Ub, OGT

*Additional sex combs like 1* (*ASXL1*) is frequently mutated in myeloid malignancies, such as MDS (myelodysplastic syndromes) as well as clonal hematopoiesis of indeterminate potential (CHIP), a disorder characterized by clonal somatic mutations only in hematopoietic cells absent other criteria for hematologic malignancies [1]. CHIP is associated with an increased risk of coronary heart disease and atherosclerosis [2], which has attracted even more attention to the *ASXL1* gene. Mutations in *ASXL1* most commonly occur as frameshift and nonsense mutations in the last exon before the C-terminal plant homeofinger domain, suggesting that the mutant mRNA escapes from nonsense-mediated decay and that most *ASXL1* mutations generate a stable truncated protein [3, 4]. Following the finding that truncated mutant forms of ASXL1 are detectable using mTRAQ-based mass spectrometric analysis [4], a growing number of mouse models with mutant expression have been developed. Recently, we generated conditional Asxl1 mutant (Asxl1-MT) knock-in mice which mimic the human E635RfsX15 mutant and found that physiological expression of Asxl1-MT *in vivo* results in modest MDS-like disease, characterized by myeloid skewing, age-dependent anemia, thrombocytosis, and morphological dysplasia [5]. Although expression of Asxl1-MT reduced the number of hematopoietic stem cells (HSCs) and stemness similarly to other MDS mice models, it maintained HSC survival in competitive transplantation assays. Moreover, it increased susceptibility to leukemic transformation caused by co-occurring *RUNX1* mutation or viral insertional mutagenesis, suggesting that these knock-in mice represent a novel model for CHIP [5].

**Figure 1:**
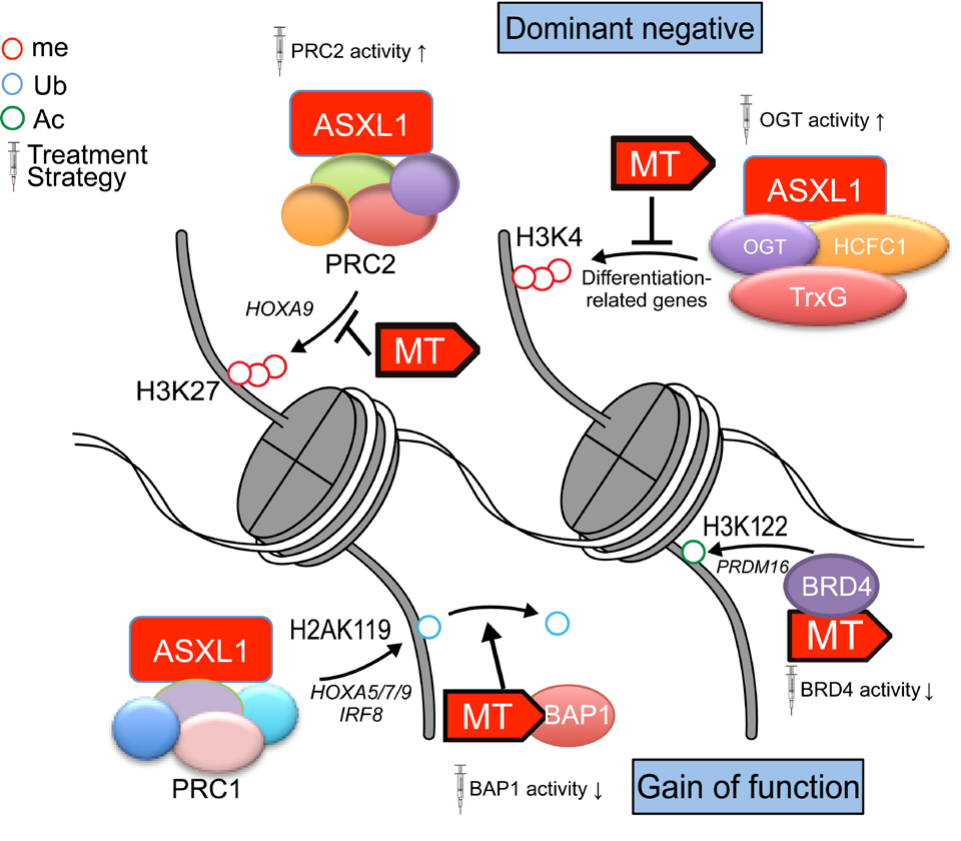
Altered histone PTM caused by ASXL1-MT and therapeutic strategies

*Asx,* the *Drosophila melanogaster* homologue of mammalian *ASXL1-3*, is required both to maintain repression and to activate expression of *Hox* genes, and *ASXL* genes are thought to mediate the balance between polycomb and trithorax functions [6], despite its lack of enzymatic activity. However, the role of the physiological expression of Asxl1-MT in histone modifications is not well understood. On ChIP-seq analysis, the Asxl1-MT knock-in mice exhibited substantial reductions in H3K4me3 and H2AK119Ub, but not in H3K27me3 [5]. Although ASXL1 interacts with PRC2 (polycomb repressive complex 2) and that loss of ASXL1 or ectopic expression of its Asxl1-MT decrease global H3K27me3 [3, 7], H3K27me3 was downregulated only at specific loci, such as posterior *Hoxa* genes, highlighting the significance of our faithfully representative model. In line with these findings, the intensity of Asxl1 wild type (WT) binding was well correlated with H3K4me3 and H2AK119Ub abundance, but not that of H3K27me3, suggesting that WT protein can support H3K4 methylation and H2AK119 ubiquitination, not H3K27 methylation. Considering that nearly all Asxl1-MT binding sites were shared with WT binding sites (93.8%), it seems that ASXL1-MT inhibits or reverses the function of WT at the same loci in terms of H3K4 and H2AK119 modification (Figure 1). In fact, the intensity of Asxl1-MT binding tends to be correlated with the reduction of H3K4me3, especially in genes associated with erythroid differentiation, which may explain the observed age-dependent anemia, a characteristic symptom of MDS.

To what extent can we explain the mechanism for the aberrant histone modification caused by Asxl1-MT? With respect to loss of H2AK119Ub, Asada et al. and Balasubramani et al. demonstrated that ASXL1-MT cooperates with BAP1 to remove H2AK119 ubiquitination [8, 9] in a gain-of-function manner. Given that WT, not MT, can bind Bmi1 [10], a component of PRC1 (polycomb repressive complex 1), and that both WT and MT interact with BAP1 [8], the C-terminus of Asxl1 may be important for the fine-tuning of H2AK119 ubiquitination. As for H3K4 methylation induced by ASXL1, we focused on the role of OGT (O-linked N-acetylglucosamine [GlcNAc] transferase)/HCFC1(host cell factor C1) based on findings from mass spectrometry and previous studies showing that the OGT/HCFC1 complex recruits trithorax homologues, such as MLL5 [11, 12]. In a recent study, we demonstrated that OGT directly stabilizes ASXL1 by O-GlcNAcylation in S199 and knockdown of ASXL1, OGT, HCFC1, or MLL5 similarly reduces global H3K4me3 and impairs hematopoietic differentiation [12]. Interestingly, there was robust overlap between H3K4me3-downregulated loci after knockdown of each component and many of the downregulated genes were involved in myeloid differentiation, splicing, and ribosomal functions, all of which are implicated in MDS pathogenesis. To determine the therapeutic potential of enhancing the ASXL1-OGT axis, we used PUGNAc, a well-studied OGA (O-GlcNAcase) inhibitor that relatively promotes OGT activity. PUGNAc clearly stabilized the ASXL1 protein and induced myeloid differentiation by upregulating *Rara* and *Egr1* with increased H3K4me3. Importantly, PUGNAc dramatically impaired the engraftment of leukemic cells with ASXL1-MT and prolonged the survival of serially transplanted mice, but this effect was not observed in the MLL-AF9 leukemia model. These data suggest that ASXL1 supports the methyltransferase activity of trithorax homologues mediated by OGT/HCFC1. Since ASXL1-WT and MT bind extensively overlapping regions of the genome [5], we speculate that ASXL1-MT may compete with WT to access DNA and to recruit H3K4 methyltransferases (Figure 1). Similarly, it is tempting to consider that replaced ASXL1-MT recruits BAP1 and releases PRC1 resulting in the deubiquitination of H2AK119 at loci which are occupied by ASXL1-WT in normal cell state. Both WT and MT seem to act as scaffolds to recruit histone modifiers, but MT counteracts WT in a dominant negative and/or gain-of-function manner, which suggests several therapeutic avenues (Figure 1).

Nonetheless, many unsolved questions remain. First, how does ASXL1 maintain a balance between active H3K4me3 marks and repressive H2AK119Ub marks? Second, the relationship between the ASXL1-OGT axis and other trithorax homologues, including MLL and SET1/COMPASS, needs to be scrutinized, as MLL5 itself lacks methyltransferase enzymatic function [13]. Notably, in a preliminary examination, we found that the localization of ASXL1-WT and MLL substantially overlap. Third, given that phosphorylation and glycosylation usually compete for the same serine/threonine residues, how does GlcNAc in ASXL1 affect other post-translational modifications (PTMs), such as phosphorylation and K351 ubiquitination, which have been shown to be involved in degradation? ASXL1 may convert external signals into epigenomic alterations via PTMs. Finally, with regard to the question of whether OGT can act against other histone modifiers and signaling pathways, hematopoietic cell-specific depletion of Ogt and S199 mutant mice will be needed to more precisely evaluate the role of ASXL1-OGT axis in normal and malignant myelopoiesis.
